# WHO recommendations on the composition of the 2017/18 influenza virus vaccines in the northern hemisphere

**DOI:** 10.2807/1560-7917.ES.2017.22.10.30479

**Published:** 2017-03-09

**Authors:** Eurosurveillance editorial team

**Affiliations:** 1 European Centre for Disease Prevention and Control (ECDC), Stockholm, Sweden

**Keywords:** influenza, influenza vaccine, influenza virus

On 2 March 2017, the World Health Organization (WHO) published recommendations on the composition of the trivalent and quadrivalent vaccines for the 2017/18 northern hemisphere influenza season [1]. WHO recommends that trivalent vaccines for use in the 2017/18 northern hemisphere influenza season contain the following:

an A/Michigan/45/2015 (H1N1)pdm09-like virus;
an A/Hong Kong/4801/2014 (H3N2)-like virus; and
a B/Brisbane/60/2008-like virus.

For the quadrivalent vaccines containing two influenza B viruses, WHO recommends that they contain the above three viruses and a B/Phuket/3073/2013-like virus.

As in previous years, national or regional authorities approve the composition and formulation of vaccines used in each country. National public health authorities are responsible for making recommendations regarding the use of the vaccine.

WHO organises consultations with an advisory group of experts twice every year to analyse influenza virus surveillance data generated by the WHO Global Influenza Surveillance and Response System (GISRS), and issues recommendations on the composition of the influenza vaccines for the following influenza season.
